# A new editor for a unique globally focused journal

**DOI:** 10.1016/j.advnut.2023.07.001

**Published:** 2023-08-01

**Authors:** Steven A. Abrams

**Affiliations:** The University of Texas Austin, USA

In August 2023 I will be honored to begin as the Editor-in-Chief of Advances in Nutrition. This journal, although relatively young, having started only 13 years ago, has had a remarkable course in becoming an important home for the highest quality review articles covering the field of nutritional sciences. Under initial Editor-in-Chief John Suttie and more recently Katherine Tucker, the journal has thrived and earned a central role in the nutrition field. It has become a place of discussion, perspective, and insight into the nutrition field of value to scientists, dietitians, physicians, and the public.

We are excited to announce that our journal will move to a monthly publication model in January 2024. This change allows us to increase both the number of journal issues published and the rate at which we present the research you conduct to the public and the scientific and medical communities. We will be introducing editorials related to a few review articles in upcoming issues. This allows an additional independent perspective on topics of importance and the field of research being reviewed.

Advances in Nutrition moved to a gold open access format in 2023. This allows its articles to be available without cost throughout the world but also requires publication fees. The open access model is a critical and positive step forward and we are excited that it enhances our goals of equity and global dissemination of nutritional research. However, we recognize the challenges this poses for some submitting authors, especially those with limited funding, and will do our best to assist authors who need assistance. Please feel free to contact me or ASN for any questions regarding our policies in this regard.

Advances in Nutrition is in excellent shape due to the hard work and skill of its editors and the American Society for Nutrition (ASN) staff to develop its mission and maintain excellence in the articles it publishes. Moving forward I hope to maintain this commitment to a high level of integrity and scientific rigor and push forward in several areas. First, it is critical that the journal serve to support diversity, equity, and inclusion in the field of nutritional science and publications of scientific research. We are committed to this and will strive to ensure that our authors, reviewers, and editors are cognizant of these perspectives and the critical importance of inclusion of all segments of the global population. Our publication will ensure a full understanding of the important cultural aspects of food and nutrition in society. We plan to broaden the editorial board and associate editor group to increase representation from differing areas of the world and population groups. We welcome articles that review issues related to nutritional diversity and public policy in support of a healthier world and social justice in ensuring safe and equitable food for all individuals.

The journal should also serve as an educational vehicle for trainees in the fields of nutrition and medicine. We encourage submissions by trainees and review of submitted articles by trainees with guidance by faculty and other supervisors. We welcome the opportunity to provide direct feedback to trainees on their submissions to enhance their interaction with the publication of science and communication of scientific findings to the public. This includes providing information via social media and podcasts as is appropriate.

By way of personal background, I am a pediatrician who is an academic faculty member at the University of Texas at Austin. I continue to practice clinical medicine as a neonatologist. My research has focused on the nutritional needs of infants and children. I am involved and committed to engaging in national and global health policy discussions regarding the critical role of nutrition in the development and health of infants and small children. I served as a member and later chair of the Committee on Nutrition of the American Academy of Pediatrics (AAP) and am co-Editor of the upcoming 9^th^ edition of the Nutrition Handbook (Yellow book) published by the AAP. I have served in a variety of federal policy roles, including being a member of the 2015 Dietary Guidelines Advisory Committee (DGAC) and now the 2025 DGAC.

Once again, I’m grateful for this opportunity to become the 3^rd^ editor-in-chief of Advances in Nutrition and would be delighted to directly speak or correspond with members of the nutrition community about the journal. You may email me at sabrams@nutrition.org with your thoughts and suggestions.

